# Multistage Segmentation of Prostate Cancer Tissues Using Sample Entropy Texture Analysis

**DOI:** 10.3390/e22121370

**Published:** 2020-12-04

**Authors:** Tariq Ali, Khalid Masood, Muhammad Irfan, Umar Draz, Arfan Ali Nagra, Muhammad Asif, Bandar M. Alshehri, Adam Glowacz, Ryszard Tadeusiewicz, Mater H. Mahnashi, Sana Yasin

**Affiliations:** 1 Department of Computer Science, Sahiwal Campus, COMSATS University Islamabad, Sahiwal 57000, Pakistan; tariqali@cuisahiwal.edu.pk; 2 Department of Computer Science, Lahore Garrison University, Lahore 54792, Pakistan; khalid.masood@lgu.edu.pk (K.M.); arfanalinagra@lgu.edu.pk (A.A.N.); drmuhammadasif@lgu.edu.pk (M.A.); 3 Electrical Engineering Department, College of Engineering, Najran University, Najran 61441, Saudi Arabia; 4 Department of Computer Science, University of Sahiwal, Sahiwal, Punjab 57000, Pakistan; 5 Department of Clinical Laboratory, Faculty of Applied Medical Sciences, Najran University, P.O. Box 1988, Najran 61441, Saudi Arabia; Bmalshehri@nu.edu.sa; 6 Department of Automatic Control and Robotics, Faculty of Electrical Engineering, Automatics, Computer Science and Biomedical Engineering, AGH University of Science and Technology, al. A. Mickiewicza 30, 30-059 Kraków, Poland; 7 Department of Biocybernetics and Biomedical Engineering, Faculty of Electrical Engineering, Automatics, Computer Science and Biomedical Engineering, AGH University of Science and Technology, al. A. Mickiewicza 30, 30-059 Kraków, Poland; rtad@agh.edu.pl; 8 Department of Medicinal Chemistry, Pharmacy School, Najran University, Najran 61441, Saudi Arabia; matermaha@gmail.com; 9 Department of Computer Science, University of Okara, Okara 56130, Pakistan; sanayaseen42@yahoo.com

**Keywords:** wavelet packets, sample entropy, mean-shift segmentation, dice coefficient

## Abstract

In this study, a multistage segmentation technique is proposed that identifies cancerous cells in prostate tissue samples. The benign areas of the tissue are distinguished from the cancerous regions using the texture of glands. The texture is modeled based on wavelet packet features along with sample entropy values. In a multistage segmentation process, the mean-shift algorithm is applied on the pre-processed images to perform a coarse segmentation of the tissue. Wavelet packets are employed in the second stage to obtain fine details of the structured shape of glands. Finally, the texture of the gland is modeled by the sample entropy values, which identifies epithelial regions from stroma patches. Although there are three stages of the proposed algorithm, the computation is fast as wavelet packet features and sample entropy values perform robust modeling for the required regions of interest. A comparative analysis with other state-of-the-art texture segmentation techniques is presented and dice ratios are computed for the comparison. It has been observed that our algorithm not only outperforms other techniques, but, by introducing sample entropy features, identification of cancerous regions of tissues is achieved with 90% classification accuracy, which shows the robustness of the proposed algorithm.

## 1. Introduction

There are around 700,000 deaths from prostate cancer per year worldwide, and it is one of the top five cancers that cause fatality [1,2]. Similar to other cancers, if prostate cancer is diagnosed at earlier stages, the chances of survival for patients are greater than 90% [3]. Experts agree that early diagnosis is key, as demonstrated by their emphasis on regular screening [4]. Screening is often targeted at high risk groups (those known to have premalignant lesions, previously treated cancer, predisposing conditions such as ulcerative colitis, or a family history) who can be followed up by episodic colonoscopy. Screening is aimed at finding polyps, a collection of tiny cells on the prostate surface that are either benign or malignant. Benign cells are not harmful, but malignant cells may develop into cancer in less than five years [5]. It is known that manual screening increases the burden on physicians, and they spend most of their time looking into benign tissues [6]. Furthermore, significant inter- and intra-observer variations in the manual diagnosis of prostate cancer have been reported in the literature [7,8]. Hence, automation in the diagnosis, especially in benign cases, will reduce the burden on pathologists, who will only spend time in examining cancer tissues and can focus more on difficult cases. Automated technologies may not provide complete tumor assessment, but they may assist the pathologists in diagnosis and providing a more accurate estimate for the prognosis [9]. Thus, it is said that histopathology is the profession that has not changed much over the last 100 years [10].

In digital pathology, digital cameras with high resolutions are used to capture the images of pathology slides. A huge amount of data is generated when digitization of glass slides is performed. There are a number of advantages for digitization that include expediate transmission, collective reporting, and forum discussions. A few challenges for digitization include lighting and staining artifacts, processing of a large amount of data and its storage, along with the absence of standards [11]. However, with the advent of advanced technologies and progress in big data and cloud computing, traditional and film-based technology is about to be eradicated and digital histopathology has replaced it completely [12].

**Figure 1:**
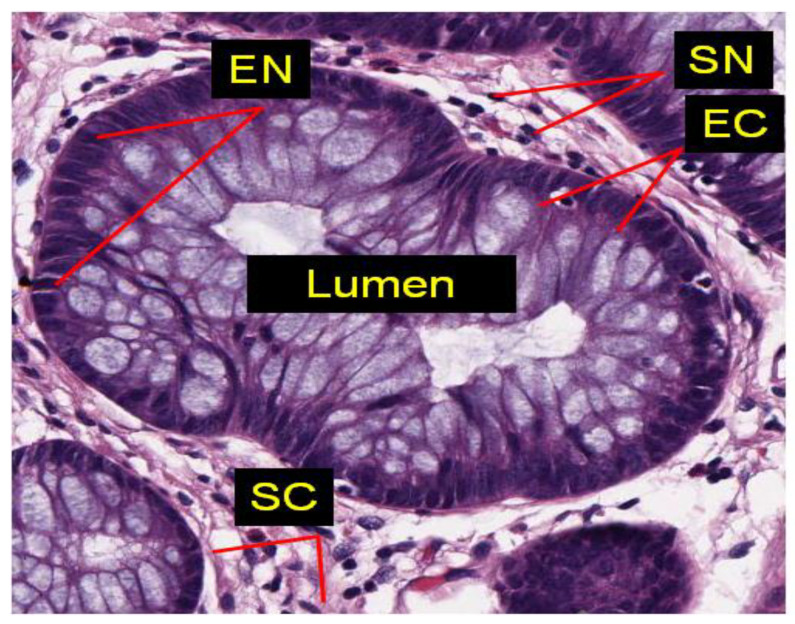
A microscopic tissue component showing various gland regions such as EN (epithelial nuclei), SN (stroma nuclei), EC (epithelial cytoplasm), SC (stroma Cytoplasm) and lumen.

In this study, we have developed an automated algorithm using texture based on sample entropy to identify cancerous tissues of prostate cancer. Texture is a feature that is determined by the spatial arrangement of the intensity levels of pixels in a closed neighborhood. It is a pattern that repeats locally for variations of the intensity of an image [13]. In a fine texture, its elements are small and variations are large, whereas in a coarse texture, its elements are large and contain several pixels. Texture can be computed by statistical, structural, or modelling methods. In our proposed technique, statistical methods using entropy and homogeneity are used for classification of benign and cancerous regions of the tissue. Sample entropy (denoted by SampEn) determines the randomness of an image and is proportional to the patterns in an image [14]. As shown in Figure 1, the tissue of a prostate gland is generally tubular in shape and its constituents are epithelial nuclei, stroma nuclei, lumen, and stroma cytoplasm. The presence of epithelial nuclei on the boundary of a prostate gland shows that the tissue structure is intact and it is benign, whereas the spread of epithelial nuclei all over the stroma and the absence of a regular shape of the gland determine that the tissue biopsy is malignant [15].

**Figure 2:**
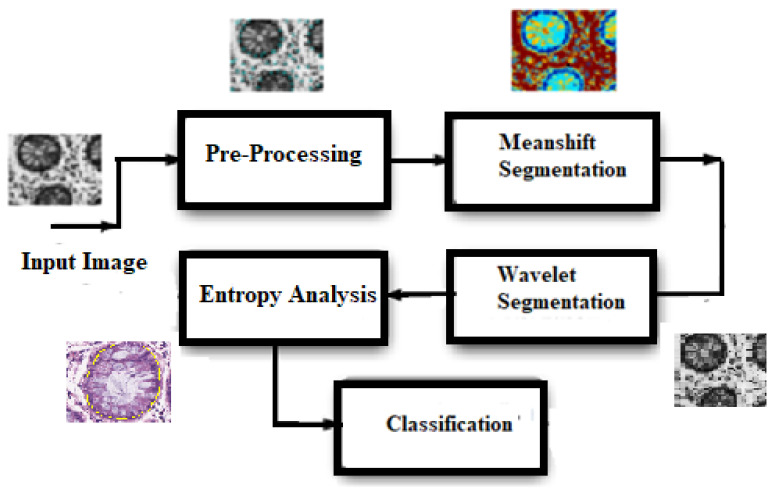
The proposed framework.

In our proposed algorithm, a multistage segmentation process for glandular structures of epithelial tissue, choosing prostate tissue as an example, is proposed. There are three stages that work in sequence to model the texture of tissue glands. After doing a few pre-processing steps, such as affine transformation and morphological operations including closing and top hat transforms, a coarse segmentation of the input images is performed using mean-shift segmentation. In the next stage, wavelet packets are used that extract glandular regions of the tissue. The texture of the extracted regions is modeled using sample entropy values and, applying a non-linear threshold, cancerous tissues are differentiated from benign tissues. The block diagram of the proposed system is presented in Figure 2.

The organization of the paper is presented as follows. Related work and background for the proposed algorithm is introduced in the next section. Section 3 presents the multistage segmentation methods of mean-shift and wavelet packets. The algorithmic framework employing the sample entropy is described in Section 4. The experimental results for segmentation of glandular structures in an experimental dataset of prostate biopsy histology images are presented and discussed in Section 5. Conclusions for the proposed model and future directions are presented in the final section.

## 2. Literature Review

A variety of algorithms have been proposed in the literature for the analysis of colon and prostate histology images (see, for example, [16,17]), but relatively few methods, such as [18,19,20], are aimed at the modeling or segmentation of glandular structures. These methods can be categorized into one of the three classes: (a) structural or texture-based segmentation, (b) boundary segmentation, and (c) region-based segmentation. Farjam et al. [21] employed variance and Gaussian filters to extract textural features from glandular structures. The authors then extract features related to size and shape of these glands and combine them to generate a malignancy index for classifying a given image into benign or malignant. However, as shown in the results, techniques that employ texture lack the prior knowledge and their performance may degrade. 

In boundary segmentation methods, Naik et al. [22] have used a level set method for active contours [23] to segment prostate tissues. The proposed technique uses a level set curve for the segmentation and employs a Bayesian classifier for the detection. Morphological features are then computed from the glandular regions and the proposed machine learning technique assigns a Gleason score to the given image. A limitation of the level set framework, however, is that the initialization of level sets may require manual intervention. Furthermore, level sets may generate errors in the case of occluded glands. Monaco et al. [24] employed a region growing algorithm to segment glands in prostate histology images to address these limitations. More recently, Monaco & Madabhushi [25] proposed a weighted version of the maximum posterior marginal estimation, designed to penalize classification errors associated with certain classes more heavily than others, and employed an extended Markov chain Monte Carlo (E-MCMC) method to detect cancerous glands in prostate histology sections.

A few regions-based approaches for glandular segmentation, such as object graphs [26] and iterative region growing [27], can also be found in the literature. The region growing algorithm of Wu et al. [28] perform segmentation for the images of intestines using adaptive thresholds for intensity values of glands. A major drawback with thresholding is that it requires manual selection, which may vary from one sample to another and is proportional to the number of samples. Moreover, the stoppage criterion for the algorithm is determined on the maximum number of iterations and the criterion varies with the number of samples in the dataset. Demir et al. [29] extended the idea of cell graphs [30] to construct object graphs for segmentation of glandular structures in colon histology images. Nuclei and lumen parts of glands are segmented using object texture information. The boundary of glands is determined using a graph-based technique that connects centroids of the nuclei. One of the limitations of this algorithm is that it adopts a semi-automatic approach to label the gland as a Boolean value to be true or false and it can be subjective. More recently, Tosun et al. [31] have proposed the idea of color graphs using color edges that carry different colors depending on the types of nodes they connect.

Nguyen et al. [32] proposed a hybrid approach involving both boundary segmentation and region growing by employing the prior knowledge about the structure of tissues for the segmentation. In the proposed algorithm, the structure of the gland is constructed by joining nuclei and cytoplasm using a technique for region growing. For pixel-level segmentation, they used the nearest neighbor classifier on the La*b* color space and classified each pixel into various classes such as epithelial nuclei, epithelial cytoplasm, lumen, and stroma. The authors also employed glandular morphological features for the purposes of Gleason grading and reported an accuracy of 88% for classifying input images into benign, grade 3, and grade 4 prostate cancer. The algorithm, however, seems to rely heavily on size constraints where there are holes in the rough glandular boundary extracted by joining nuclei and cytoplasm. Because of the separation of boundary extraction and region growing, the algorithm does not take into account the very intimate spatial relationship between the epithelial boundary of glandular structures and lumen texture of the inside of glandular regions. 

In the work of [33], a stack of five convolution neural network (CNN) layers is used to determine prostate cancer. An automatic process was adopted to generate region of interests (ROIs), and this is both the advantage and limitation of the proposed algorithm. There is no ground truth, so the validation lacks the input from an expert. The algorithm performs superiorly with an 84% area under the curve. In [34], the transfer learning approach using deep learning was used to classify prostate cancer. The deep learning approach using CNN was compared with various machine learning algorithm such as decision tree, support vector machine (SVM), and Naive Bayes, and it was found that the CNN outperforms all the machine learning techniques. The limitation of the approach is that transfer learning is employed, which normally suffers from overfitting.

## 3. Materials and Methods

Modeling of the glandular structures in the proposed algorithm, as shown in Figure 2, is described briefly as follows:

(1) As a first step, segmentation of the tissue is achieved by applying the color mean-shift (MS) algorithm [34] on the input image I(z). The tissue is divided into four parts that contain epithelial nuclei, cytoplasm, lumen, and stroma nuclei.
(2) The above segmentation result can be used to get a rough idea of pixels forming nuclei, both stromal and epithelial. In the second step, textural features for pixels of the image are used to model the glands. The wavelet packet features [35] and AdaBoost classifier are used in assigning lumen-ness labels T(z) (1 for lumen, and 0 for non-lumen) for all pixel locations z ϵ Z in the image I(z).
(3) Finally, the texture of the image is modeled using sample entropy analysis. Using empirical methods, a threshold is obtained that distinguishes cancerous regions form the benign tissue samples.

**Figure 3:**
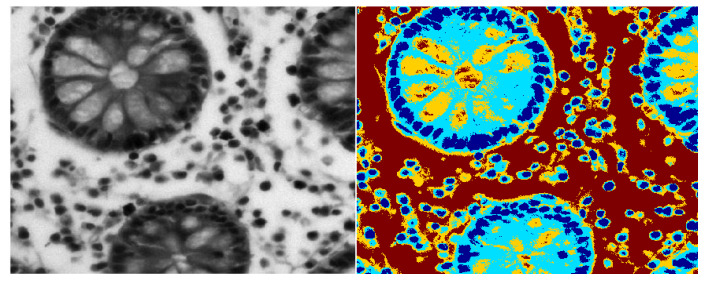
Mean shift segmentation for benign circular glands.

**Figure 4:**
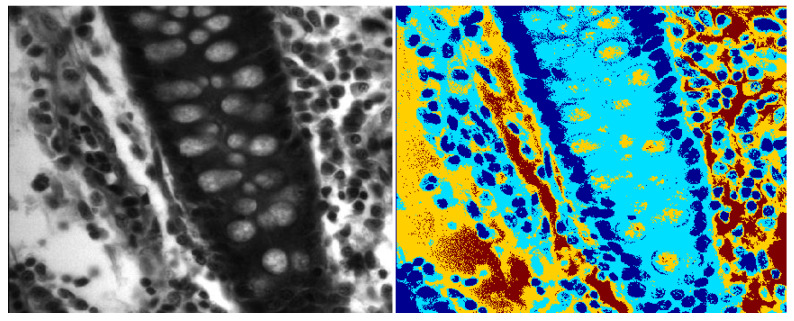
Mean shift segmentation for benign elliptical glands.

Our hypothesis is that glandular structures are formed by epithelial nuclei at the boundary of glands and the lumen is inside them. We employed the color mean-shift (MS) algorithm [36] for a coarse segmentation of prostate tissue into its four constituents that contain epithelial/stromal nuclei, cytoplasm, lumen, and stromal background. The color MS algorithm is an unsupervised clustering algorithm that assigns each pixel the weighted mean of its neighboring pixels, where the weight assigned to each of the neighboring pixels is given by a Gaussian kernel, which determines the photometric similarity between a pixel z under consideration and all pixels in its neighborhood N(z). Iterative application of the color-based MS algorithm results in a coarse segmentation of the tissue types, as shown in Figure 3 and Figure 4. The MS algorithm performs an initial segmentation, followed by the fine segmentation using wavelet packets.

In wavelet packets, both low and high frequency components are decomposed in each sub-band. An analogy of binary trees is presented for wavelet packets where every low or high sub-band represents a node with two children. As the binary tree may have either 0 or 2 children, the basis of wavelet packets is 2^2n^, where n is the level of decomposition. In the wavelet packet analysis, the image is examined at different levels. The over-complete wavelet packet decomposition provides a feature vector that is computed from the energy and entropy features of the texture that is obtained by wavelet packets and sample entropy analysis. In wavelet packet decomposition, the approximate and detail images at each level are decomposed into four levels and a quad tree image filter is achieved. In conventional wavelet packets transform, the down-sampled sub images are one-quarter of the size of the original image. However, in over-complete filter decomposition, there is no down-sampling and the sub-bands are of the same size as the original. Thus, a robust feature vector is obtained. A high number of features can be obtained with an increase in the decomposition level, as the number of sub-bands increases exponentially. In two-level decomposition, eight feature sub images are retained only and these images contain maximum variance in each level.

In sample entropy, the spatial distributions of pixels are correlated to compute the entropy distributions [37]. The entropy is an irregularity measure of the texture in images. It determines the similarity of two patterns k and k + 1, if their pixel distribution is alike. The similarity is based on the distance (d) of each corresponding pixel in both of the patterns. The probability is computed based on the pattern matches in k and k + 1 pattern. The probability of k + 1 patterns of the same distribution is determined by the ratio of the similarity match in k patterns. In computing the sample entropy, each k length pattern is tested for all k length patterns in the entire image. A match is recorded if each pixel on k + 1th pattern differs by not more than distance (d) from the corresponding pixels in k window (pattern). Thus, sample entropy reflects the repeated-ness of patterns in images. The low values of entropy show more regular texture and fine details, whereas high values of entropy reflect irregular texture with coarse representation.

Sample entropy is a measure of irregularity and periodic or regular patterns show small values of sample entropy. Let us consider two patterns *P^m^*(d) and *P^m^*^+1^(d); by determining their logarithmic ratio, the sample entropy can be computed. An image I (*i*, *j*) with *n_i_* rows and *n_j_* columns is considered that contains *x_k_* (*i*, *j*) set of pixels varying from *j* to *j* + *m* − 1 column lines and *i* to *i* − *m* + 1 row. The window size is *x_k_* (*i*, *j*) and it is centered at *i* and *j*. There are only (*n_i_* − *k*) × (*n_j_* − *k*) patterns as the last m lines cannot be used for square patterns as the total number of pixels is equal to *N*, where *N* = *n_i_* × *n_j_*. To determine the mathematical formulation of the sample entropy, the following derivations are used.

$$SEn=-ln\frac{P^{m+1}\left(r\right)}{P^{m}\left(r\right)}$$

where

$$P^{m}\left(r\right)=\frac{1}{N_{p}}\overset{N_{p}}{\underset{i=1}{\sum }}P_{i}^{m}\;P^{m+1}\left(r\right)=\frac{1}{N_{p}}\overset{N_{p}}{\underset{i=1}{\sum }}P_{i}^{m+1}$$

where r represents the number of grey levels and *N_p_* is the total number of m length different squared patterns in an image, whereas *P^m^* and *P^m^*^+1^ are m and *m* + 1 length pattern in the image, respectively. The sample entropy is the logarithmic ratio for these patterns. The entropy and energy are computed for each segmented sub image with the square local area obtained by sample entropy window size. Energy is the content of information in an image. It is defined on the normalized histogram of an image and it shows how gray levels are spread in the image. The energy of an image is inversely proportional to Shannon entropy and is closely related to randomness. A high energy image has a low number of gray levels. For each squared area, the feature vector represents values for the central pixel of the sub image.

## 4. Results

We use texture as a cue for assigning lumen-ness labels to all pixels in the image. The wavelet packet transform is used to compute pixel-based features. In the wavelet transform, the approximation filter is not divided and only the last level is split for the high levels of approximation and detailed images. The idea behind the wavelet packet transform is to further decompose both approximation as well as detail sub-bands at each but the last level. The advantage is that useful information in the high signal wavelengths is computed for the richer analysis of textural features. The wavelet packet features perform fine segmentation of the tissue. AdaBoost is added in the model that enhances the performance of the classifier. In AdaBoost modeling, weak classifiers are converted into a strong classifier.

Daubechies 4 filters of wavelet packets are used to extract a 64-dimensional feature vector. Pixel labeling algorithm using wavelet packet features is trained on 50 random samples in the training data. There were 25 prostate cancer (suspected) patients and 2 samples were collected from each patient. We trained and tested the proposed technique on a dataset of 50 Hematoxylin and Eosin (H&E) stained prostate histopathology images. The dataset is collected from National Cancer Institute (NCI), where regions of interest (ROIs) from whole slide images are extracted at 40X magnification [38]. The resolution of each image is 2048 × 2048. For the ground truth, manual segmentation was performed by the pathologist who assisted in extracting the ROIs as well. There was one expert who marked all the ground truths (GTs). However, the digitization of the ground truth samples was sent to our collaborator in the other institute, who endorsed most of the GT samples. The difference in opinion was for only two samples, which was later approved and accepted by the expert and the GTs for the two samples were updated accordingly. The Dice similarity coefficient was used to evaluate the performance of our proposed algorithm that computes the area of overlap for automated segmentation with the ground truth images.

An average patch size of 50 × 50 pixels was selected such that half of them were from lumen areas and the other half were from the stromal areas for each of the training images. The wavelet packet transform for the third level generates features for the energy and entropy of each patch and a 64-dimensional feature vector is produced. The Adaboost classifier is used to enhance the accuracy of SVM. The Adaboost technique enhances the performance of a weak classifier, which has accuracy of slightly more than guessing, into a strong classifier. In each iteration of the Adaboost algorithm, the weight of the misclassified observation is increased, otherwise the weight is reduced. In a few iterations, the training error is reduced exponentially, and the algorithm achieves rapid convergence. The trained classifier is then used to assign pixel labels to each image pixel in our dataset based on their 64-dimensional wavelet packet features.

## 5. Discussion

**Figure 5:**
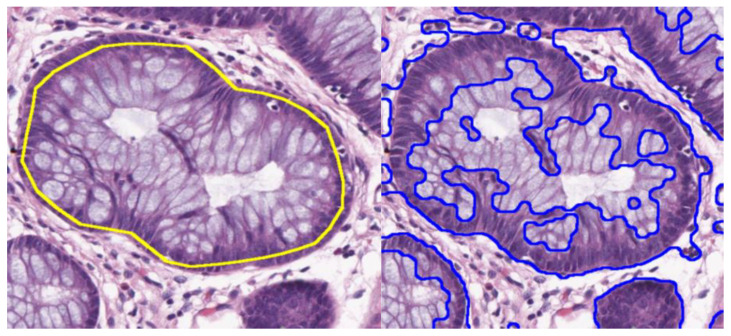
Comparison of the Gland segmentation by the proposed algorithm (yellow boundary line) and Gaussian filtering algorithm (GFT) by [39] (blue boundary lines).

To test the quality of the segmentation results by our algorithm, ground truth glandular regions are computed for each gland marked with its boundary. In Figure 5, the segmented image of the proposed algorithm (TPA) is compared with the Gaussian filtering technique (GFT) in [39]. A major strength of our algorithm is that it does not result in holes, formed during segmentation of the lumen when only texture is used as a cue, because of its use of prior knowledge about gland formation and sequentially connecting epithelial nuclei on the glandular boundary. The classification criterion is based on the sample entropy and wavelet packet features for a patch. The low values of the sample entropy represent a uniform structure and, by computing an empirical threshold, a tissue is considered to be normal if the values for a patch are less than the threshold, and it can be in various stages of cancer if the patch values are above the determined threshold.

**Figure 6:**
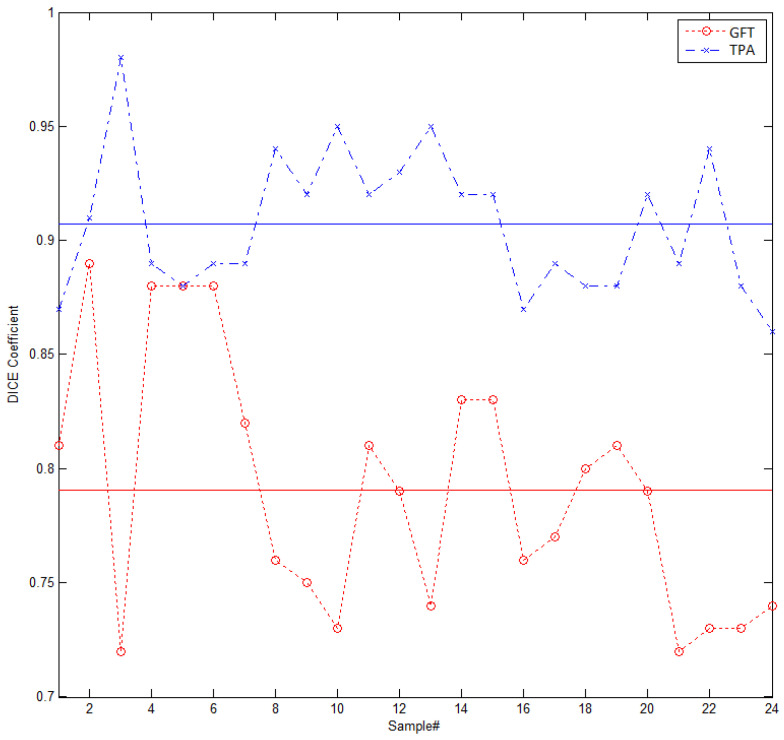
Dice similarity coefficient (DSC) for the proposed algorithm (TPA) and Gaussian filtering technique (GFT) in [39] for the first 24 samples in our dataset. The average DSC for our technique is 0.91, whereas for GFT, it is 0.79.

To evaluate the performance of our algorithm, the Sorensen–Dice index or Dice Similarity Coefficient (DSC) is used as a quality measure with its value ranging between 0 and 1 [40]. It is a similarity index between two sets of samples. The Dice similarity coefficient (DSC) of zero shows the least agreement between automated segmentation and the ground truth, while a value approaching one represents good agreement between the two. The DSC is computed as 2 times the area of overlap divided by the total number of pixels in both images as in our case, one is the automated segmented image and the other is the ground truth image. The DSC is the same as the F1 score, which is computed as follows:

DICE = (2 × *TP*)/(*TP* + *TP* + *FP* + *FN*)

where *TP* represents total number of positive samples that are also positive in the ground truth, whereas *FP* is the false positive cases, which are false in the ground truth, but predicted to be true by the algorithm. *FN* represent the false negatives that the algorithm predicts to be negative, while in the ground truth, they are positive. Figure 6 presents a comparison of the segmentation accuracy of the two algorithms, GFT of [39] and the proposed algorithm (TPA) using DSC. As shown in Figure 6, the proposed model always produces better results than the Gaussian filtering-based segmentation, with average DSC coefficient values being 0.79 and 0.91 for [39] and our method, respectively.

To evaluate the experimental results, we have computed quality metrics such as accuracy, precision, and recall. They are defined as follows:

$$Accuracy=\frac{\left(TP+TN\right)}{\left(TP+TN+FP+FN\right)}$$

$$Precision\;=\frac{TP}{\left(TP+FP\right)}$$

$$Recall\;=\frac{TP}{\left(TP+FN\right)}$$

The parameters in the confusion matrix are explained as follows.

- True-positive (*TP*): The algorithm detects it as cancerous and the ground truth also labels it as cancerous.
- False-positive (*FP*): The algorithm detects it as cancerous, while the ground truth labels it as normal.
- True-negative (*TN*): The algorithm detects it as normal, whereas ground truth also labels it as normal.
- False-negative (*FN*): The algorithm detects it normal, whereas ground truth labels it as cancerous.

Table 1 presents the classification attributes of samples in our experimental dataset. The texture of tissues is modeled using sample entropy values. The support vector machine (SVM) classifier using a Gaussian kernel is employed to separate the two classes of data samples. The model achieves 90% classification accuracy, which supports the notion that sample entropy values contain discriminative features that separate benign samples from malignant samples. However, a large-scale validation of these results is required for endorsing the results achieved by the proposed algorithm.

## 6. Conclusions

In this study, our proposed algorithm identifies cancerous regions of prostate histology images using the multi-segmentation technique. The model performs two-step segmentation for prostate tissue images. Initially, the MS (mean-shift) algorithm is used to perform the coarse segmentation to split the tissue constituents in four parts. In the second stage, wavelet filters are used to perform fine segmentation of the tissue glands. For classification, sample entropy features are extracted from the segmented regions along with the energy parameters. It has been observed that sample entropy achieves more than 90% classification accuracy. An added advantage of performing multistage segmentation using sample entropy values is that one could easily separate epithelial nuclei from the stroma nuclei in standard H&E stained images without using any additional immunohistochemical (IHC) markers. Furthermore, morphological signatures of glandular structures modeled using our algorithm can also be fused with disease signatures from other modalities such as mass spectrometry (MS), IHC, flow cytometry (FC), and/or cytogenetic (CG) testing for integrated diagnostic/prognostic purposes [41,42]. Future directions of this work include extension of this model for application to whole-slide histology images of other epithelial cancers such as colon and lung. Moreover, additional data samples will be added for large-scale validation of the model. For robust and efficient utilization of the proposed model, autoencoders with zero shot learning that do not require large training samples will be incorporated into the classification algorithm.

## Figures and Tables

**Table 1:** Quality metrics for classification of tissue regions into benign and malignant samples. SVM, support vector machine.

Quality Metrics	Benign	Malignant
	SVM (%)	SVM (%)
Accuracy	90	91
Precision	86	89
Recall	81	84
F1 Score	85	87
